# Systemic Lupus Erythematosus Presenting With Thrombotic Thrombocytopenic Purpura at Onset: A Case Report

**DOI:** 10.3389/fped.2022.931669

**Published:** 2022-08-01

**Authors:** Yoko Takagi, Yasuko Kobayashi, Ayako Hirakata, Mariko Takei, Satoshi Ogasawara, Chikage Yajima, Yuka Ikeuchi, Akira Matsumoto, Yoshiyuki Ogawa, Hiroshi Handa, Masanori Matsumoto, Hirokazu Arakawa, Takumi Takizawa

**Affiliations:** ^1^Department of Pediatrics, Gunma University Graduate School of Medicine, Maebashi, Japan; ^2^Department of Hematology, Gunma University Graduate School of Medicine, Maebashi, Japan; ^3^Department of Blood Transfusion Medicine, Nara Medical University, Kashihara, Japan

**Keywords:** systemic lupus erythematosus, thrombotic microangiopathy, thrombotic thrombocytopenic purpura, ADAMTS13 activity, anti-ADAMTS13 antibody, platelet thrombosis, von Willebrand factor, lupus nephritis

## Abstract

**Background:**

Thrombotic microangiopathy (TMA) is a syndrome associated with hemolytic anemia, thrombocytopenia, and various organ disorders. Thrombotic thrombocytopenic purpura (TTP) is a disease that develops when a disintegrin-like and metalloproteinase with thrombospondin type l motif 13 (ADAMTS13) activity decreases to < 10% of that in normal plasma, causing platelet thrombosis in microvessels throughout the body. Currently, ADAMTS13-deficient TMA is diagnosed as TTP. Systemic lupus erythematosus (SLE)-related TMA includes both acquired TTP, in which ADAMTS13 activity is significantly reduced, and secondary TMA, in which ADAMTS13 activity is not reduced. Both diseases have different prognoses.

**Case Presentation:**

An 11-year-old girl was admitted to our hospital on suspicion of TMA with thrombocytopenia and hemolytic anemia. Because the patient had hypocomplementemia, SLE-related TMA or complement-related TMA was considered. Therefore, we initiated plasma exchange (PE) for the patient. Subsequently, she fulfilled the pediatric SLE diagnostic criteria, and ADAMTS13 activity was shown to be decreased and the anti-ADAMTS13 antibody titer increased. She was thus diagnosed with acquired TTP caused by SLE. Treatment response was good as a platelet count and ADAMTS13 activity improved with three times of PE, followed by methylprednisolone pulse therapy and administration of mycophenolate mofetil. Renal pathology showed thrombus formation in glomerular arterioles and lupus nephritis categorized as Class III (A) of the International Society of Nephrology and the Renal Pathology Society classification. Because the patient was thought to be in the high-risk group of SLE, three courses of intravenous cyclophosphamide pulse therapy were administered as an additional induction therapy. No recurrence of TTP was observed.

**Conclusion:**

In SLE-related TMA, measurement of ADAMTS13 activity and the anti-ADAMTS13 antibody titer are necessary for diagnosis, and for predicting prognosis and recurrence of the disease; however, in the acute phase of immune-mediated TMA, it is important to initiate proper treatments even before knowing the results to improve prognosis.

## Introduction

Thrombotic microangiopathy (TMA) is a syndrome associated with hemolytic anemia, thrombocytopenia, and various organ disorders (1). Collagen diseases have been reported to be the most common underlying cause of secondary TMA, among which systemic lupus erythematosus (SLE) is the most common (2).

Thrombotic thrombocytopenic purpura (TTP), a typical example of TMA, is caused by a deficiency in enzymatic activity of a disintegrin-like and metalloproteinase with thrombospondin type l motif 13 (ADAMTS13), which induces platelet aggregation and systemic microvascular thrombosis (3). Since ADAMTS13 activity became measurable in clinical practice, reduction of its activity to < 10% of that in normal plasma has become one of the diagnostic criteria for TTP. SLE-related TMA includes acquired TTP, in which ADAMTS13 activity is reduced to < 10%, and secondary TMA, in which ADAMTS13 activity is ≥ 10%. The prognoses of the two conditions are significantly different (4). In addition, anti-ADAMTS13 antibody titer measurement is important to diagnose acquired TTP in SLE; however, this process is time intensive. Because TMA is life-threatening in the acute phase, physicians are occasionally forced to decide therapeutic strategy prior to obtaining results on ADAMTS13 activity and the anti-ADAMTS13 antibody titer.

Herein, we report a pediatric case of acquired TTP with the concurrent onset of SLE. We experienced difficulties in managing a platelet count until the ADAMTS13 activity and anti-ADAMTS13 antibody titer were confirmed. Plasma exchange (PE) and methylprednisolone pulse therapy (MPT) were quite effective, but prednisolone and plasma infusion were insufficient to improve a platelet count. In addition, typical renal pathology in TTP was observed with occlusive thrombosis in afferent glomerular arteriole in the present case (5).

## Case Presentation

An 11-year-old girl had gross hematuria from the morning 2 days before admission and a slight fever of 37°C since that night. On the next day, she visited her home doctor and was revealed to have thrombocytopenia and hemolytic anemia (platelet count, 13,000/μL; hemoglobin, 9.7 mg/dL; lactase dehydrogenase, 1,046 U/ml, with 83 schistocytes out of 1,000 erythrocytes counted), in addition to hematuria and proteinuria. The patient was transferred to our hospital with suspected TMA. Her height and weight were 142 cm [–0.6 standard deviation (SD)] and 30.8 kg (–1.0SD), respectively. Her vital signs on admission were as follows: body temperature, 37.9°C; heart rate, 105 bpm; blood pressure, 122/80 mmHg; SpO_2_, 98% with room air. The patient appeared pale and ill, although she was alert. Petechia was observed on her soft palate. She had a normal breath sound and no heart murmur. Her abdomen was soft and flat without tenderness. Purpura was scattered on her upper arms, lower legs, and precordium on both sides. No edema was found on her lower legs. She had no past medical history and no family history of blood diseases, autoimmune diseases, or renal diseases. Laboratory findings on admission are shown in Table 1. The data available by the end of the first hospital day revealed that her hemolytic anemia and thrombocytopenia were accompanied by hypocomplementemia. Direct Coombs test result was negative. Kidney function was normal despite abnormality in urinalysis. Although the patient did not complain of gastrointestinal symptoms or bloody stool, stool culture, verotoxin in her stool, and anti-O157 antibody in her serum were tested to exclude hemolytic uremic syndrome (HUS) caused by enterohemorrhagic *Escherichia coli* infection; all test results were negative. Finally, we found that ADAMTS13 activity was decreased to < 1% and the anti-ADAMTS13 antibody titer returned positive.

**TABLE 1:** Laboratory findings on admission.

**<Blood Count>**		**<Biochemical Exam>**		Ca	9.0 mg/dL	RF	<11 IU/mL	**<Urinalysis>**	
Hematocrit	25.3%	TP	6.9 mg/dL	Ferritin	1109 ng/dL	Anti-Sm Ab	29.3	Color	Brown
Hemoglobin	8.8 g/dL	Albumin	4.5 g/dL	Glucose	76 mg/dL	Anti-SS-A Ab	119.9	pH	6.0
RBC	307 × 106/μL	T-Bil	4.28 mg/dL	T-Chol	220 mg/dL	Anti-SS-B Ab	Negative	Proteinuria	4+
WBC	4300/μL	D-Bil	0.35 mg/dL	TG	108 mg/dL	Anti-RNP Ab	Negative	Hematuria	3+
Platelet	0.8 × 10^3^/μL	AST	238 U/L			Anti-ds-DNA Ab	179 IU/mL	Glucose	-
Schistocyte	83/1000	ALT	171 U/L	**<Serological Exam>**		PR3-ANCA	<1.0 U/mL	Ketone	2+
		LDH	1431 U/L	CRP	0.25 mg/dL	MPO-ANCA	<1.0 U/mL	WBC	-
**<Hemostasis>**		ALP	929 U/L	Haptoglobin	<8 mg/dL	LA	Negative	RBC	35/HPF
PT	108%	CK	78 U/L	CH50	≤14 U/mL	Anti-CL Ab	Negative	Epitherial cast	<1/LPF
PT-INR	0.97	BUN	25 mg/dL	C3	70 mg/dL	O-157 LPS Ab	Negative	Granular cast	<1/LPF
APTT	27.6 s	Creatinine	0.54 mg/dL	C4	5.0 mg/dL			Hyaline cast	<1/LPF
Fibrinogen	308 mg/dL	eGFR	94.3 mL/min/1.73 m^2^	IgG	1048 mg/dL	Direct coombs	Negative	Pro/Cr	8.34 g/g Cre
FDP	11.1 μg/mL	Na	138 mEq/L	IgA	118 mg/dL	Blood type	O Rh(+)		
D-dimer	4.3 μg/mL	K	4.0 mEq/L	IgM	62 mg/dL			**<Fecal Test>**	
		Cl	103 mEq/L	ASLO	94 IU/mL	ADAMTS13 act	<1 %	Culture	Negative
				ANA	80×	Anti-ADAMTS13 Ab	1.4 BU/mL	Verotoxin	Negative

*Ab, antibody; Act, activator; ALP, alkaline phosphatase; ALT, alanine aminotransferase; ANA, antinuclear antibody; Anti-CL Ab, anti-cardiolipin antibody; anti-ds-DNA Ab, anti-double-stranded DNA antibody; anti-RNP Ab, anti-ribonucleoprotein antibody; anti-Sm Ab, anti-smooth muscle antibody; APTT, activated partial thromboplastin time; ASLO, anti-streptolysin-O; AST, aspartate aminotransferase; BUN, blood urea nitrogen; C3, complement 3; C4, complement 4; CH50, 50% hemolytic unit of complement; CK, creatine kinase; CRP, C-reactive protein; D-Bil, direct-bilirubin; eGFR, estimated glemerular filtration rate; FDP, fibrinogen degradation products; IgG, immunoglobulin G; IgA, immunoglobulin A; IgM, immunoglobulin M; LA, lupus anticoagulant; LDH, lactate dehydrogenase; MPO-ANCA, myeloperoxidase-anti-neutrophil cytoplasmic antibodies; O-157 LPS Ab, O-157 Lipopolysaccharide antibody; PR3-ANCA, proteinase-3-anti-neutrophil cytoplasmic antibodies; Pro/Cre, protein creatinine ratio; PT, prothrombin time; PT-INR, prothrombin time-international normalized ratio; RBC, red blood cells; RF, rheumatoid factor; T-Bil, total-bilirubin; T-Cho, total-cholesterol; TG, triglyceride; TP, total protein; WBC, white blood cells.*

*Underlined values indicate abnormality.*

**FIGURE 1:**
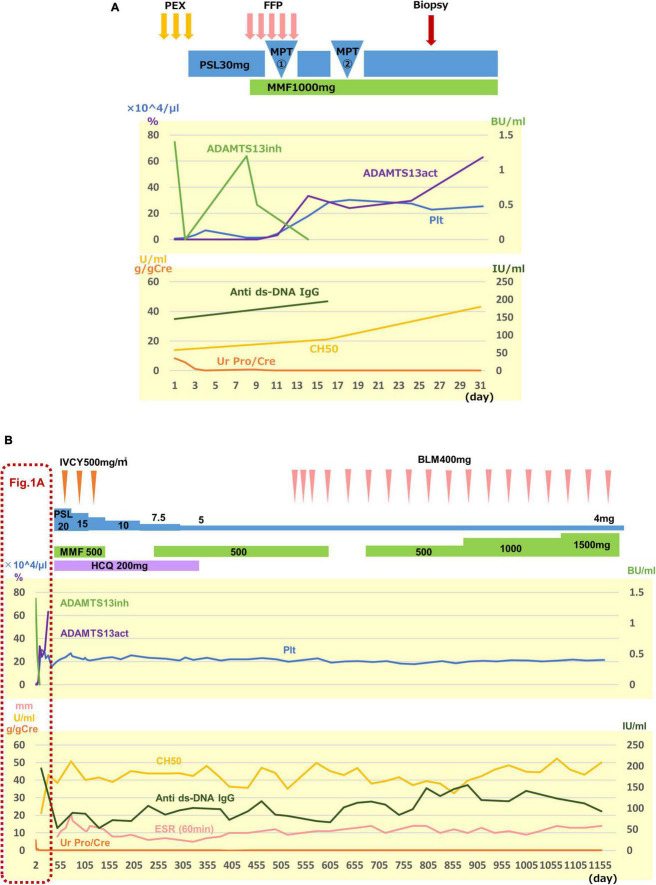
Clinical course. The upper graph shows the activity of TTP, and the lower graph shows the disease activity of SLE and lupus nephritis, both in (A,B). (A) Clinical course for the first 31 days after admission is shown. Three courses of plasma exchange (PE) were effective to remove anti-ADMTS13 antibody (ADMTS13inh) and to increase a platelet count (Plt) rapidly. However, 30 mg of prednisolone (PSL) injection and fresh-frozen plasma (FFP) infusion were not sufficient to suppress antibody production to recover ADAMTS13 activity (ADAMTS13act) and to maintain the platelet count. After two courses of methylprednisolone pulse therapy (MPT), the recovery of the ADAMTS13 activity and the platelet count was observed. Proteinuria disappeared after the first course of pulse therapy. Renal biopsy was performed on the 25th hospital day. (B) Overall clinical course is shown. Treatment details and clinical data are shown following (A). Since the patient was considered to be in the high-risk group of SLE organ damage, and TMA is known to have frequent complications of psychiatric symptoms, she was additionally treated with three courses of intravenous cyclophosphamide pulse therapy (IVCY) as induction therapy for SLE, in addition to mycophenolate mofetil (MMF) and hydroxychloroquine (HCQ). HCQ was discontinued because color blindness and retinopathy were suspected at a regular ophthalmologic visit, and belimumab (BLM) was started instead just after BLM had become under health insurance coverage for SLE pediatric patients (B). Ur Pro/Cre, urinary protein creatinin ratio; anti ds-DNA IgG, anti-double-stranded DNA immunogloburin G; ESR, erythrocyte sedimentation rate.

The clinical course is shown in Figure 1. After admission, PE was performed for 3 days for TMA, which successfully resulted in a rapid platelet count increase up to 71,000/μL, and PE was completed. Since she fulfilled the SLE diagnostic criteria, including positive antinuclear antibody and anti-ds-DNA antibody on the 3rd hospital day, prednisolone (1mg/kg) was initiated. On the 7th hospital day, her platelet count decreased to 10,000/μL. This led to the consideration of PE again; however, the patient was too distressed to undergo PE. Therefore, we first attempted to administer fresh-frozen plasma, which was found to be insufficient for a significantly improving platelet count. Meanwhile, we obtained the results of ADAMTS13 activity and anti-ADAMTS13 antibody, as described in Table 1. The patient was thus diagnosed with acquired TTP caused by SLE, and MPT was administered on the 10*^th^* hospital day. After two courses of MPT, the anti-ADAMTS13 antibody test result was negative, the ADAMTS13 activity increased, and the platelet count rapidly increased (Figure 1A). Additionally, urinary findings were normal after the first course of pulse therapy. Mycophenolate mofetil (MMF, 1,000 mg/day) and hydroxychloroquine (HCQ, 200 mg/day) were administered for SLE. Since she was considered to be in the high-risk group of SLE organ damage, and TMA is known to have frequent complications of psychiatric symptoms (4), she was additionally treated with three courses of intravenous cyclophosphamide pulse therapy [IVCY, 580 mg/dose (500 mg/m^2^)] as induction therapy for SLE (Figure 1B). After the three courses of IVCY, leukopenia, mainly in lymphocytes, was observed; therefore, MMF was discontinued temporarily. HCQ was discontinued because color blindness and retinopathy were suspected during a regular ophthalmologic visit. After 1 year and 4 months since the onset, belimumab (BLM) was administered instead just after BLM had become under health insurance coverage for SLE pediatric patients (Figure 1B).

**FIGURE 2:**
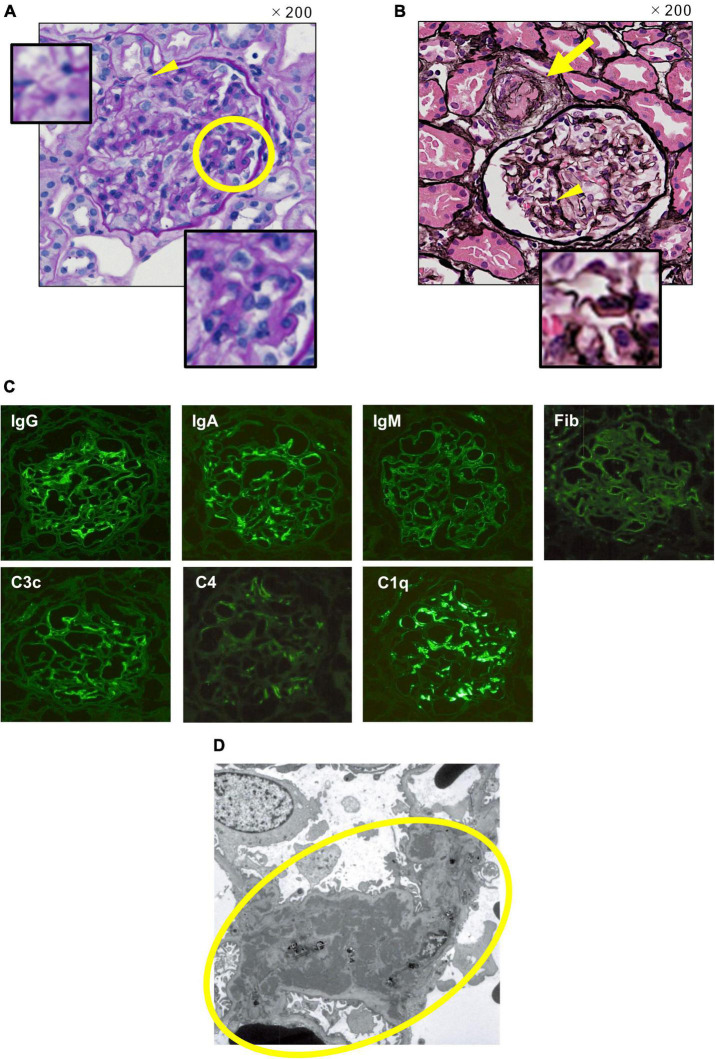
Renal pathology. (A) Light microscopy. Periodic acid Schiff stain, original magnification 200 ×. A wire loop lesion and endocapillary hypercellularity (the yellow circle), and duplication in glomerular basement membrane (the yellow arrowhead) are observed. (B) Light microscopy. Periodic acid silver-methenamin + hematoxylin-eosin staining, original magnification 200 ×. Thrombus formation with luminal stenosis in afferent arteriole (the yellow arrow) and wire loop lesion (the yellow arrowhead) are observed. (C) Immunofluorescent staining. Full-house positive staining at the capillary wall and, mainly, in the paramesangial region is shown. (D) Electronic microscopy. Electron-dense deposits in the paramesangial region (the yellow circle) are observed comparable to IF findings.

Renal biopsy was performed on the 25*^th^* hospital day. Light microscopic findings in the renal pathology showed focal and segmental mild subendothelial deposition, basement membrane duplication, and endothelial cell proliferation. The findings led to a pathological diagnosis of lupus nephritis International Society of Nephrology and the Renal Pathology Society (ISN/RPS) Classification III (A) (Figure 2A). Notably, granular periodic acid-Schiff stain-positive thrombi were found in afferent arterioles, and glomeruli with luminal stenosis in afferent arterioles were observed in 6 of 26 glomeruli, which was considered to be consistent with TTP (Figure 2B). Immunofluorescent staining (IF) showed full house positive at the capillary wall and, mainly, in the paramesangial region (Figure 2C). Electron-dense deposits were observed in the paramesangial region with electron microscopy, which were comparable to IF findings (Figure 2D).

## Discussion

Herein, a pediatric case of acquired TTP with the concurrent onset of SLE is described. In TTP, platelet thrombosis in microvasculature causes severe damage to kidneys, brain, and other end organs due to low ADAMTS13 metalloprotease activity (3). ADAMTS13 specifically cleaves von Willebrand factor (VWF) into various size multimers, which are mainly produced in vascular endothelial cells. Uncleaved large VWF multimers, due to the low ADAMTS13 activity, bind to platelets with high affinity and form platelet thrombi under high fluid shear stress in microvessels, resulting in subsequent thrombosis in patients with TTP (6, 7).

To diagnose a patient with hemolytic anemia and thrombocytopenia of unknown etiology, ADAMTS13 activity is a key test in the diagnostic process of TTP, after excluding HUS caused by Shiga toxin-producing *E. coli* infection (3). Patients in whom ADAMTS13 activity is reduced to < 10% are diagnosed with TTP. Among patients with TTP, those negative for anti-ADAMTS13 antibody are diagnosed with congenital TTP, such as Upshaw–Schulman syndrome (USS), and those positive for the antibody are diagnosed with acquired TTP. On the other hand, if ADAMTS13 activity is ≥ 10%, atypical HUS (aHUS) with complement abnormality or secondary TMA with underlying conditions is considered in the differential diagnosis. In the present case, the patient was found to fulfill the SLE diagnostic criteria. Therefore, acquired TTP or secondary TMA was considered in relation to SLE. In addition, aHUS and even USS had been considered in the differential diagnosis, until the ADAMTS13 activity was revealed to have decreased to < 10%. Since anti-ADAMTS13 antibody was reported positive, the patient was diagnosed with acquired TTP and USS was excluded. Generally, SLE is complicated with both acquired TTP with decreased ADAMTS13 activity and secondary TMA without decreased ADAMTS13 activity. Thus, the ADAMTS13 activity test is important for the differential diagnosis of SLE-related TMA.

Yue et al. reported that the incidence of TMA in patients with SLE was 3% (62 of 2,062 patients with SLE), and the incidence of acquired TTP was 0.29% (6 of 2,062 patients with SLE) (4). Sato et al. reported that 5 (2.2%) of 222 patients with SLE were diagnosed with TTP with four or five of the classic pentad of TTP, and no patient fulfilled the recent criteria for acquired TTP with < 10% of ADAMTS13 activity among the SLE cohort (8). Reese et al. identified 79 acquired TTP with a severe reduction in ADAMTS13 activity in children from a systematic literature review. Of the 79 patients, 10 (12.6%) were described to have SLE (9). Fujimura and Matsumoto reported that collagen disease-related TMA accounted for 24% (221 out of 919) of patients with TMA and was the most common underlying disease in acquired secondary TMA, according to the Japanese TMA registry from 1998 to 2008. SLE was the most common disease (41%, 92 out of 221 collagen disease-related TMA) among them (2).

Matsuyama et al. analyzed clinical characteristics and plasma ADAMTS13 levels in 127 patients with collagen disease-related TMAs, including 64 patients with SLE. Half of the patients with SLE-related TMA have a subnormal to normal range (> 25%) of ADAMTS13 activity, and 23.4% of the patients with SLE-related TMA have a severe deficiency (< 5%) of ADAMTS13 activity. Furthermore, 32 of the patients with SLE-related TMA were ADAMTS13 inhibitor-positive. The group with severe ADAMTS13 deficiency responded well to treatments, and the prognosis was rather favorable (10). Yue et al. compared two groups of patients with SLE-related TMA: one group included 6 patients with acquired TTP secondary to SLE, and the other included 13 patients with SLE-related TMA negative for the ADAMTS13 inhibitor and whose ADAMTS13 activities were within the normal range. They concluded that the TTP group is associated with more severe thrombocytopenia, central nervous system involvement, mild renal involvement, rapid resolution, and relatively good treatment response (4).

Plasmapheresis is the only treatment that has been scientifically shown to be effective in the treatment of acquired TTP (11). Since it has been reported that delayed PE worsens prognosis (12), PE should be initiated as soon as possible when acquired TTP is suspected. Currently, as ADAMTS13 activity measurement result takes several days in daily clinical practice, treatment should be initiated even without results. Steroid therapy is also recommended with the expectation of suppressing autoantibody production (13). Both steroid pulse therapy and high-dose steroids are administered, although it is unclear which therapy is more efficacious (13). In our case, as shown in Figure 1, the platelet count rapidly increased after three courses of PE and two courses of MPT after the anti-ADAMTS13 antibody became negative and the ADAMTS13 activity increased in response to the treatments. Thrombus formation in afferent arterioles in glomeruli with luminal stenosis was still observed in renal pathology (Figure 2) obtained on the 25^th^ hospital day, indicating the severity of organ damage in the acute phase of the disease.

In conclusion, measurement of ADAMTS13 activity and the anti-ADAMTS13 antibody titer is necessary for the diagnosis and proper treatment of SLE-related TMA; however, in the acute phase of immune-mediated TMA, it is important to initiate PE even before knowing the results to improve prognosis. Pediatric SLE-related TTP cases need to be accumulated to elucidate appropriate treatment and to improve long-term prognoses as this is a rare condition, and clinical features of pediatric SLE are partially different from adult SLE.

## Patient Perspective

It’s already been three years since I was discharged from the hospital. At first I wasn’t sure what happened to me, and I really don’t remember anything from when I was in the ICU. When I moved from the ICU to the pediatric ward, I was worried when I heard from my family that my condition was pretty bad, but with the support of doctors, nurses, other patients in the same room, and my family, now, I am here. All the children in the same room were nice, and each was having a hard time with their own disease. I realized that for a child fighting illness, every single day in the hospital is a battle against time and their body. I am very grateful to my family who accompanied me in the hospital and to the doctors and pharmacists who helped me with the treatment and medicine.

## Data Availability Statement

The original contributions presented in this study are included in the article/supplementary material, further inquiries can be directed to the corresponding author.

## Ethics Statement

Ethical review and approval was not required for the study on human participants in accordance with the local legislation and institutional requirements. Written informed consent to participate in this study was provided by the participants or their legal guardian/next of kin.

## Author Contributions

YT and YK wrote the manuscript and performed the practical work. AH, MT, SO, CY, and YI collected clinical data. AM, YO, HH, and MM contributed to measuring ADAMTS13 activity and anti-ADAMTS13 antibody sequentially and to analyzing the data. HA and TT revised the manuscript. All authors contributed to the article and approved the submitted version.

## Conflict of Interest

The authors declare that the research was conducted in the absence of any commercial or financial relationships that could be construed as a potential conflict of interest.

## Publisher’s Note

All claims expressed in this article are solely those of the authors and do not necessarily represent those of their affiliated organizations, or those of the publisher, the editors and the reviewers. Any product that may be evaluated in this article, or claim that may be made by its manufacturer, is not guaranteed or endorsed by the publisher.

## Abbreviations

aHUS: atypical HUS
ADAMTS13: a disintegrin-like and metalloproteinase with thrombospondin type l motifs 13
BLM: belimumab
HCQ: hydroxychloroquine
HUS: hemolytic uremic syndrome
IF: immunofluorescent staining
ISN/RPS: International Society of Nephrology and the Renal Pathology Society
IVCY: intravenous cyclophosphamide pulse therapy
MMF: mycophenolate mofetil
MPT: methylprednisolone pulse therapy
PE: plasma exchange
SD: standard deviation
SLE: systemic lupus erythematosus
TMA: thrombotic microangiopathy
TTP: thrombotic thrombocytopenic purpura
VWF: von Willebrand factor
USS: Upshaw–Schulman syndrome.
